# Recent Developments in Atomic Layer Deposition of Functional Overlayers in Perovskite Solar Cells

**DOI:** 10.3390/nano13243112

**Published:** 2023-12-10

**Authors:** Helen Hejin Park, David J. Fermin

**Affiliations:** 1 Advanced Materials Division, Korea Research Institute of Chemical Technology (KRICT), Daejeon 34114, Republic of Korea; 2 Department of Advanced Materials and Chemical Engineering, University of Science and Technology (UST), Daejeon 34113, Republic of Korea; 3 School of Chemistry, University of Bristol, Bristol BS8 1TS, UK

**Keywords:** metal halide perovskite, solar cell materials, atomic layer deposition, photovoltaics

## Abstract

Over the last decade, research in organic–inorganic lead halide perovskite solar cells (PSCs) has gathered unprecedented momentum, putting the technology on the brink of full-scale commercialization. A wide range of strategies have been implemented for enhancing the power conversion efficiency of devices and modules, as well as improving stability toward high levels of irradiation, temperature, and humidity. Another key element in the path to commercialization is the scalability of device manufacturing, which requires large-scale deposition of conformal layers without compromising the delicate structure of the perovskite film. In this context, atomic layer deposition (ALD) tools excel in depositing high-quality conformal films with precise control of film composition and thickness over large areas at relatively low processing temperatures. In this commentary, we will briefly outline recent progress in PSC technology enabled by ALD tools, focusing on layers deposited above the absorber layer. These interlayers include charge transport layers, passivation layers, buffer layers, and encapsulation techniques. Additionally, we will discuss some of the challenges and potential avenues for research in PSC technology underpinned by ALD tools.

## 1. Introduction

The most abundant resource available to humanity, solar energy, has been extensively investigated for decades, leading to a technology learning curve of 20%. Indeed, powerful data-driven energy–technology–economy simulations developed by Nijsse et al. have shown conclusive evidence that photovoltaics (PV) has already passed the technology tipping point and are set to dominate the global energy market by 2060 [1]. Researchers are exploring a variety of PV technologies with a shared common goal: the cost-effective and efficient harnessing of solar energy for decarbonizing human activity. Organic–inorganic hybrid perovskite solar cells (PSCs) have rapidly emerged as a promising technology, characterized by a fast increase in conversion efficiency and low-cost fabrication methods, bringing them close to the threshold of commercial viability.

While PSCs have demonstrated remarkable progress in power conversion efficiency, achieving an impressive 26.1% for unit cells [2], they present unique challenges compared to other PV technologies. These challenges are predominantly linked to long-term device stability, which is determined by internal and external factors. Internal factors involve issues such as ion migration in the perovskite and the diffusion of additives from hole transport layers into the perovskite. On the other hand, external factors encompass device degradation caused by exposure to elevated temperatures, high irradiation levels, and their high sensitivity to humidity and oxygen [3,4,5,6,7,8,9,10,11,12,13,14].

High performance at the module scale remains a formidable challenge for the successful commercialization of PSCs. In addition to device stability, other technology bottlenecks include efficiency drop in large-area devices, scalable manufacturing techniques, as well as material toxicity. These efforts will be pivotal in bringing PSCs closer to becoming a practical and sustainable solar energy solution.

Atomic layer deposition (ALD) is an effective and versatile tool for producing pinhole-free, uniform, reproducible, and high-quality inorganic thin films. ALD’s strength lies in its ability to precisely control the thickness of the film and tailor material properties, such as morphology, doping, and stoichiometry [15,16,17]. With the capacity for large-scale deposition at low temperatures, ALD has proven pivotal in a range of applications, spanning from microelectronics to large-scale energy technologies such as batteries and PV. 

As summarized in Table 1, ALD has emerged as an attractive tool for depositing device components, from passivating to charge transporting layers (CTL), leading to significant improvement in performance. However, the deposition of ALD thin-film overlayers is far from trivial due to the susceptibility of organic transport layers and perovskite films to precursors, temperatures, and vacuum conditions required in ALD. Naturally, when employing ALD layers prior to the deposition of the perovskite layer in single absorber devices, there are significantly fewer restrictions in the process parameters. This commentary discusses recent advances in the deposition of functional thin films onto PSC absorbers by ALD, highlighting current challenges and opportunities this tool can offer.

## 2. ALD Films Deposited above Active Layers

### 2.1. Charge Transport Layers

**Figure 1:**
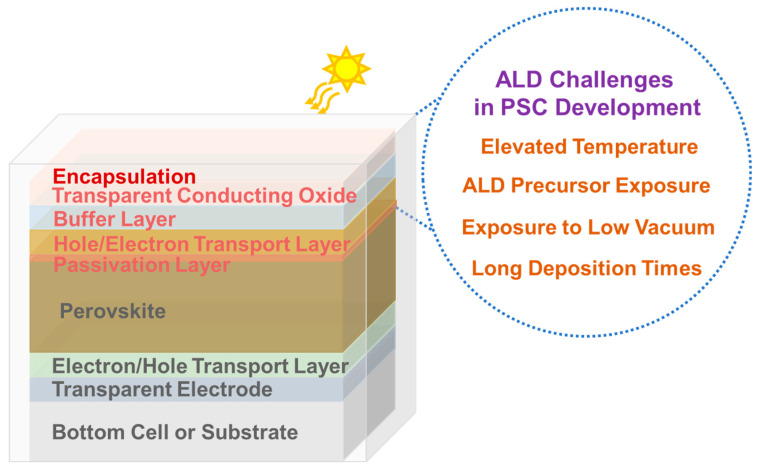
Challenges in incorporating ALD are PSCs for layers above the perovskite.

As illustrated in Figure 1, challenges associated with employing ALD overlayers stem from the vulnerabilities of the perovskite material and organic hole-transport layers (HTL) to environmental factors. These challenges include the sensitivity to exposure to specific ALD precursors (including H_2_O), moisture, thermal energy, and prolonged exposure to low vacuum conditions during the deposition process.

**Figure 2:**
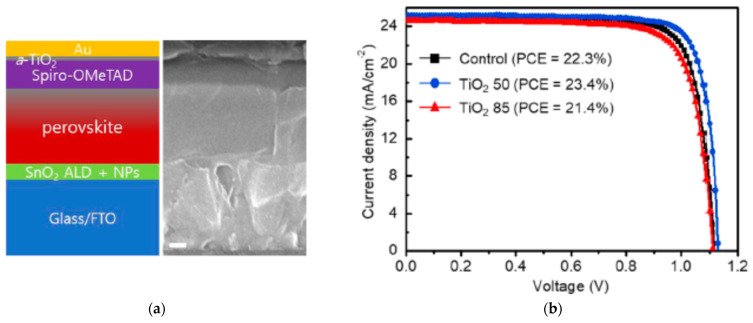

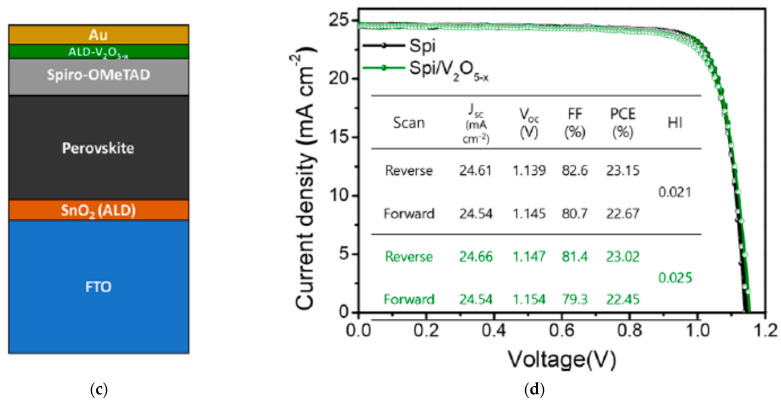
Incorporation of ALD processes for TiO_2_ and V_2_O_5−*x*_ in PSCs: (**a**) schematic of solar cell device stack and cross-sectional scanning electron microscopy image of PSC device; (**b**) illuminated *J*-*V* scans comparing PSCs without and with ALD TiO_2_. Reproduced with permission from [18]. American Chemical Society, 2021. (**c**) Schematic of solar cell device stack of PSC device; (**d**) illuminated *J*-*V* scans comparing PSCs without and with ALD V_2_O_5−*x*_. Reproduced with permission from [19]. American Chemical Society, 2022.

As exemplified in Figure 2, one of the strategies in which ALD overlayers have had a strong impact is in interlayers located between the HTL and top contact. Amorphous titanium dioxide (*a*-TiO_2_) [18] and vanadium oxide (V_2_O_5−*x*_) [19] by ALD have been inserted above spiro-OMeTAD further improving the photovoltaic performances. Improvement in photovoltaic device performance parameters from ALD interlayer insertion above the absorber is summarized in Table 1. In these cases, processing conditions should be carefully tuned to minimize the impact of temperature and precursor gases on the active layers. As discussed in the next section, the introduction of additional protective layers capable of shielding the active layers from direct exposure to the ALD process [20] has yielded significant improvement in device performance.

**Table 1:** Summary of recent literature on ALD interlayers inserted above the absorber in perovskite solar cells.

Material	Device Stack	*J_SC_* (mA/cm^2^)	*V_OC_* (V)	*FF* (%)	*η* (%)	Institute, Year [Ref]
Al_2_O_3_	FTO/*c*-TiO_2_/*mp*-TiO_2_/FAPbI_3_/Al_2_O_3_ (<1 nm)/OAI/spiro-OMeTAD/Au	25.2 → 25.2	1.10 → 1.15	80.0 → 83.6	22.2 → 24.1	KRICT, 2023 [21]
Al_2_O_3_	FTO/SnO_2_/MAPbI_3_/OLAI/spiro-OMeTAD/Au/Al_2_O_3_ (36 nm)	21.9 → 22.6	1.08 → 1.15	76.8 → 81.0	18.2 → 20.9	IIT Bombay, 2023 [22]
Al_2_O_3_	ITO/*c*-TiO_2_/MAPb(I_1−*x*_Cl*_x_*)_3/_Al_2_O_3_ (1 nm)/spiro-OMeTAD/Au	21.3 → 21.7	1.03 → 1.07	69.0 → 77.0	15.1 → 18.0	Eindhoven, 2017 [23]
CuO*_x_*	FTO/*c*-TiO_2_/*mp*-TiO_2_/FA_0.95_MA_0.05_Pb(I_0.95_Br_0.05_)_3_/PTAA/pulsed-CVD CuO*_x_* (15 nm)/ITO	21.7	1.01	71.1	15.6	KRICT, 2020 [20]
CuO*_x_*	FTO/*c*-TiO_2_/*mp*-TiO_2_/Cs_0.05_(MA_0.17_FA_0.83_)_0.95_Pb(I_0.83_Br_0.17_)_3_/PTAA /AP-CVD CuO*_x_* (3 nm)/ITO/MgF_2_	20.6	1.10	73.7	16.7	Cambridge, 2020 [24]
Ga_2_O_3_	FTO/Li:NiO/MAPbI_3_/IDIC/PCBM/BCP /Ga_2_O_3_ (<2 nm)/Ag	22.4	1.12	79.4	19.9	Wuhan, 2018 [25]
SiAl*_x_*O*_y/_*SiO_2_	ITO/SnO_2_/(FAPbI_3_)_0.85_(MAPbBr_3_)_0.15_/PTAA/SiAl*_x_*O*_y_*/SiO_2_/Au	22.0 → 22.6	1.12 → 1.14	69.0 → 75.0	17.1 → 19.2	NCEPU, 2023 [26]
SnO*_x_*	ITO/2PACz/Perovskite-Wide/LiF/C_60_/SnO*_x_*/Au/PEDOT:PSS/Perovskite-Narrow/C_60_/BCP/Ag	14.8 → 15.2	1.94 → 2.01	77.5 → 77.6	22.3 → 23.7	NREL, 2023 [27]
SnO_2_	Si PV/ITO/NiO*_x_*/Perovskite/C_60_/SnO_2_/IZO/Ag	19.01	1.85	75.7	26.7	Nankai U., 2022 [28]
SnO_2_	ITO/PTAA/Perovskite/GABr/PCBM/BCP/ SnO_2_ (30 nm)/Cu	21.7 → 21.9	1.16 → 1.19	76.2 → 81.1	19.2 → 21.1	Nankai U., 2022 [29]
SnO_2_	Si PV/ITO/PTAA/ Cs_0.15_(FA_0.83_MA_0.17_)_0.85_Pb(I_0.7_Br_0.3_)_3_/ICBA/C_60_/ SnO_2_/IZO/MgF_2_	17.8	1.80	79.4	25.4	UNC, 2019 [30]
SnO_2_	Si PV/spiro-TTB/Cs*_x_*FA_1−*x*_Pb(I_1−*y*_Br*_y_*)_3_/ LiF/C_60_/**SnO_2_**/IZO/MgF_2_	19.5	1.74	74.7	25.4	EPFL, 2019 [31]
SnO*_x_*/Zn:SnO*_x_*	ITO/PTAA/Cs_0.05_FA_0.80_MA_0.15_Pb (I_0.85_Br_0.15_)_3_/C_60_/BCP/**SnO*_x_*** (6 nm)/Zn:SnO*_x_* (2 nm)/IZO	20.8	1.12	79.3	18.5	NREL, 2019 [32]
*a*-TiO_2_	FTO/SnO_2_/FAPbI_3_/spiro-OMeTAD/TiO_2_ (5 nm)/Au	24.9 → 24.9	1.08 → 1.11	79.1 → 80.2	21.3 → 22.3	SKKU, 2021 [18]
V_2_O_5-*x*_	FTO/SnO_2_/FA_0.95_Cs_0.05_Rb_0.01_PbI_3_/spiro-OMeTAD/V_2_O_5−_***_x_*** (5 nm)/Au	24.6 → 24.7	1.14 → 1.15	82.6 → 81.4	23.2 → 23.0	SKKU, 2022 [19]
VO*_x_*	ITO/*np*-SnO_2_/C_60_/FA_0.83_MA_0.17_Pb(I_0.83_Br_0.17_)_3_/ spiro-TTB/VO*_x_* (9 nm)/ITO	18.9	1.07	71.0	14.2	Stanford, 2019 [33]
ZrO_2_	FTO/NiO*_x_*/*e*-MoO*_x_* (10 nm)/MAPbI_3_/ZrO_2_ (<2 nm)/PC_61_BM/Al	21.5 → 21.9	1.01 → 1.11	75.0 → 75.0	16.3 → 18.2	SCN, 2018 [34]

### 2.2. Passivation Layers

In cases where passivation layers are located directly above the perovskite absorber, thickness is a crucial parameter, often limited to 1 nm or less [21,23]. In these cases, the exposure time of the perovskite material to ALD precursor gases, thermal energy, and the vacuum environment is usually confined to approximately 10 min. This limited exposure minimizes the potential damage that the ALD process may cause to the perovskite absorber. Ultra-thin films deposited under these conditions are likely to be amorphous, and their electron transport properties can be a complex convolution of parameters, including the chemical nature of the precursors [35].

**Figure 3:**
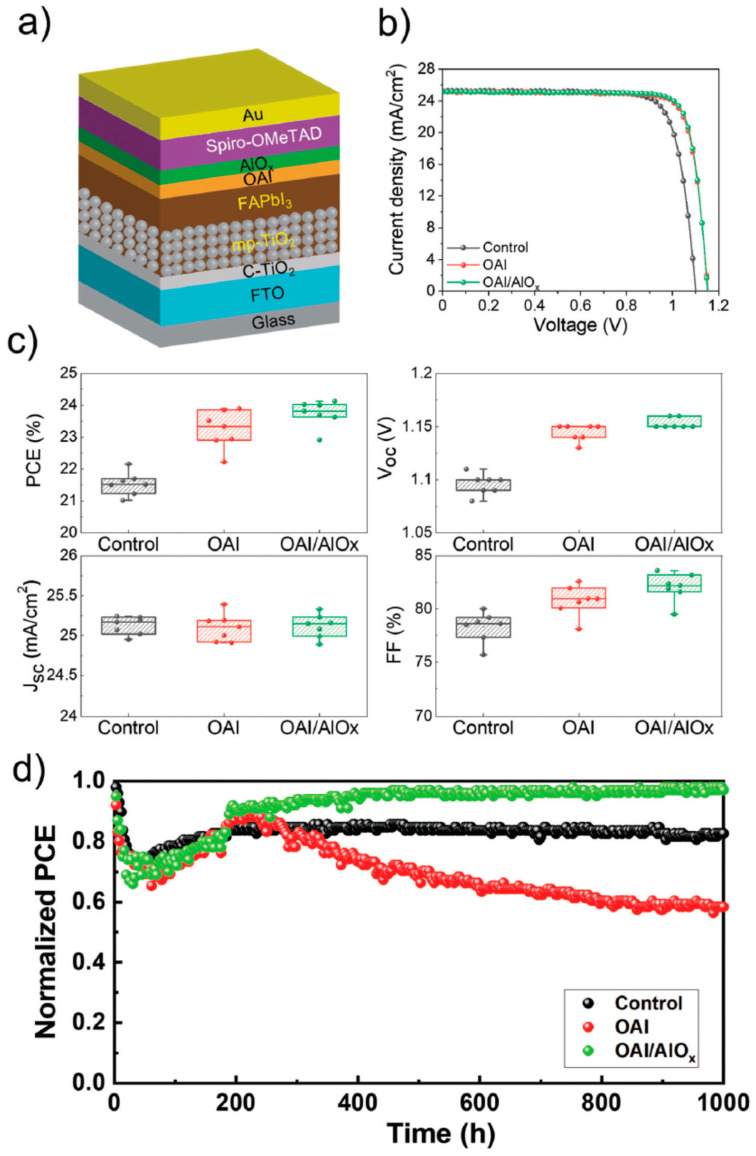
Incorporation of ALD AlO*_x_* in combination with octylammonium iodide (OAI) in PSCs: (**a**) schematic of solar cell device stack of PSC device; (**b**) illuminated *J*-*V* scans comparing PSCs with OAI and OAI/ALD AlO*_x_*. (**c**) Photovoltaic device performance parameters based on the different passivation treatments; (**d**) device stability measurements of encapsulated devices under 1 SUN with maximum power point tracking. Reproduced with permission from [21]. Wiley, 2023.

Ultra-thin films of less than 1 nm are deposited between the perovskite absorber and the CTL, as exemplified in Figure 3. This layer may not only enhance the performance of solar cell devices by improving parameters such as fill factor (FF) and open-circuit voltage (*V_OC_*) but also contribute significantly to the stability of the device [36]. The observed enhancements in operational stability can be attributed to two key mechanisms: the surface passivation of the perovskite and the creation of a barrier separating the absorber from the CTL.

While analogous surface passivation concepts have been successfully demonstrated by generating a two-dimensional perovskite layer on the surface of a three-dimensional perovskite layer using solution processing [37], researchers have also explored the effectiveness of various barrier layers created via ALD. These ALD-deposited barrier layers exhibit notable improvements in device stability when exposed to moisture and light. Specifically, several research groups have employed ALD to create barrier layers utilizing insulating materials such as zirconium oxide (ZrO_2_) and aluminum oxide (Al_2_O_3_) [21,23,34].

Introducing an ultra-thin passivation layer of Al_2_O_3_, measuring less than 1 nm in thickness, between the perovskite layer and HTL under the so-called *n*-*i*-*p* device architecture has yielded notable enhancements in device performance. This innovation has led to improved open-circuit voltage and fill factor [23]. The Al_2_O_3_ passivation layer not only boosted the power conversion efficiency of the PSC but also mitigated hysteresis effects and bolstered the device’s resilience against high humidity. X-ray diffraction (XRD) confirmed the structural integrity of Al_2_O_3_ passivated methylammonium lead iodide (MAPbI_3_) films exposed to humidity, while non-passivated films revealed the emergence of a PbI_2_ (001) under identical conditions [23]. Additionally, ongoing photovoltaic performance assessments under humid conditions confirmed the superior stability of PSCs containing the Al_2_O_3_ passivation layer.

A recent study investigated the impact of combining perovskite surface passivation with octylammonium iodide (OAI) and ALD AlO*_x_* [21]. While the introduction of OAI on the perovskite layer yielded enhancements in device performance, it was noted that the light stability and resistance to damp heat conditions diminished when compared to unpassivated perovskite devices. However, when ALD AlO*_x_* was introduced after OAI on the perovskite layer, a different outcome was observed. This dual approach not only improved device performance but also enhanced the light stability and damp heat stability of the devices, as shown in Figure 3. This improvement can likely be attributed to the diffusion of aluminum from AlO*_x_* into the perovskite, which contributes to uniform photo-generated carrier transport, both at the surface and within the bulk of the material. Additionally, this process leads to the formation of light-induced two-dimensional perovskite structures. These structural changes play a role in preventing the loss of octylammonium cations due to the presence of AlO*_x_*, resulting in a reduction in the number of iodine anions. This reduction, in turn, helps suppress light-induced degradation in the perovskite, ultimately enhancing the stability of the devices.

Exploring the impact of ZrO_2_ passivation has yielded positive results, leading to enhanced power conversion efficiencies via improved *V_OC_* values in *p*-*i*-*n* devices. In the case of PSCs based on MAPbI_3_, the addition of the ZrO_2_ passivation layer between the perovskite and ETL resulted in a *V_OC_* enhancement of 0.1 V. Meanwhile, PSCs based on methylammonium lead bromide (MAPbBr_3_) exhibited an even more substantial *V_OC_* improvement of 0.5 V with the incorporation of the ZrO_2_ insertion. Furthermore, the stability of both device types, without and with ZrO_2_, displayed significant enhancements, underscoring the overall enhancement in device stability [34].

At the interface between the CTL and the top metal contact, passivation or protective measures can also be applied. In this context, between the electron transport layer (ETL) and the top metal contact, silver (Ag), a thin layer (measuring less than 2 nm) of a wide bandgap material, gallium oxide (Ga_2_O_3_), was introduced using ALD [25]. One of the well-known degradation mechanisms in perovskite solar cell devices is the formation of AgI due to the diffusion of Ag and iodine ions, which leads to a decline in device performance over time. The inclusion of Ga_2_O_3_ acts as a stabilizing factor, preventing the formation of AgI. This Ga_2_O_3_ protective layer acts as a barrier, protecting against moisture ingress and hindering the corrosion process at the interface between the top Ag electrode and the device. Moreover, the introduction of this protective layer serves to reduce carrier recombination, lower current leakage, and enhance the quality of interfacial contact. Overall, the Ga_2_O_3_ protection layer plays a substantial role in improving PSC performance and durability.

S. Ghosh et al. reported that the process of spiro-OMeTAD coating on perovskite forms buried defect states, which are detrimental to device stability [22]. Passivation of these buried defect states was shown to be possible by depositing 36 nm of ALD Al_2_O_3_ on top of fully functional devices. Such passivation technique resulted in an increase in efficiency mainly due to improvement in *V_OC_* by ~60–70 mV and enhanced device stability under MPPT under ambient and even high vacuum conditions.

### 2.3. Buffer Layers in Tandem and Semitransparent Applications

Tandem and semitransparent architectures necessitate a semitransparent top electrode to replace the opaque metal one. The prevailing technique for creating transparent electrodes in such applications involves employing sputtered transparent conducting oxides (TCOs), such as indium zinc oxide (IZO) and indium tin oxide (ITO). However, this approach typically necessitates the inclusion of a buffer layer beneath the TCO to shield the underlying organic layer from sputtering damage during the TCO deposition process.

In *p*-*i*-*n* architectures for tandem applications, the commonly used sputter buffer layers include tin oxide (SnO_2_) [30] or a combination of SnO_2_ and zinc tin oxide (ZTO) [32], generally deposited by ALD. The introduction of these buffer layers not only protects the CTL but also optimizes the band alignment at the buffer/TCO interface.

For semitransparent *n*-*i*-*p* perovskite solar cells, molybdenum oxide (MoO*_x_*) by thermal evaporation has traditionally served as the conventional buffer layer. However, MoO*_x_* suffers from poor stability in the presence of air [38]. To address this limitation, alternative buffer layers have been explored, including copper oxide (CuO*_x_*) and vanadium oxide (VO*_x_*), both deposited via ALD in semitransparent PSCs [20,33]. Innovative growth methods such as atmospheric-pressure chemical vapor deposition (AP-CVD) [24] and pulsed-chemical vapor deposition (pulsed-CVD) [20] have been reported for CuO*_x_* buffer layers in semitransparent *n*-*i*-*p* PSCs. Notably, AP-CVD CuO*_x_* films demonstrated high carrier mobilities exceeding 4 cm^2^/V·s and achieved impressive power conversion efficiencies exceeding 16% when incorporated into semitransparent devices [24].

### 2.4. Encapsulation

To shield perovskite solar cells from external environmental influences like oxygen and moisture, encapsulation is an essential requirement. Numerous studies have highlighted effective encapsulation techniques for PSC devices, employing single materials or nanolaminates created via ALD or incorporating organic materials. For instance, in the case of semitransparent PSC devices, a successful encapsulation strategy involved employing a 50-nanometer bilayer of PET (polyethylene terephthalate) coated with Al_2_O_3_. This approach resulted in durable devices that remained stable when stored in ambient air for a period exceeding 45 days [39].

## 3. Deposition Process Parameters and ALD Equipment

ALD process parameters, such as precursors and deposition temperature, and equipment information of the studies covered in this commentary article are summarized in Table 2. Most of the deposition temperatures are kept below 120 °C. Trimethylaluminum (TMA), bis(1-dimethylamino-2-methyl-2-butoxy)copper(II) (Cu(dmamb)_2_), allyloxytrimethylsilyl hexafluoroacetylacetonate copper(I) (ATHFAACu), tris(dimethylamino)gallium (Ga_2_(NMe_2_)_6_), tetraethyl orthosilicate (TEOS), tetrakis(dimethylamido)tin(IV) (TDMASn), diethylzinc (DEZ), tetrakis(dimethylamido)titanium(IV) (TDMATi), vanadium(V) tri-*i*-propoxyoxide (VTIP), and tetrakis(dimethylamide) zirconium(IV) (TDMAZr) were used as the aluminum, copper, copper, gallium, silicon, tin, zinc, titanium, vanadium, and zirconium precursors, respectively. Mostly, deionized water was used as the oxygen precursor. However, there were studies also using hydrogen peroxide (H_2_O_2_) and ozone (O_3_).

## 4. Challenges in Implementing ALD on PSC Device Processing and Alternative Approaches

While ALD offers numerous advantages, including precise control over stoichiometry and thickness with exceptional reliability, it is important to note that for certain layers, particularly those exceeding 15 nm on top of the perovskite absorber, prolonged exposure to specific ALD precursors, elevated temperatures, and low vacuum conditions can have adverse effects on the organic charge transport layer and/or the perovskite [40]. In perovskite solar cells, most ALD processes above the absorber are ideally carried out at low temperatures (typically below 100 °C) to minimize thermal-induced stress.

Concerning damage resulting from exposure to ALD precursors, some studies have indicated a reduction in bending and stretching modes of N-H groups with increasing ALD cycles of Al_2_O_3_, as observed via in situ infrared spectroscopy. This suggests the potential loss of nitrogen from etching the methylammonium (MA^+^) cations in the perovskite lattice [39]. Consequently, deviations from conventional ALD methods are often required to reduce exposure to degradation sources and minimize deposition time.

Alternative techniques, such as atmospheric-pressure chemical vapor deposition (AP-CVD) [24], pulsed-chemical vapor deposition (pulsed-CVD) [20], and spatial ALD (s-ALD) [41,42], have been employed to address these challenges. Pulsed-CVD, for example, involves pulsing the ALD precursors simultaneously rather than separately and reducing the purging step during the ALD sequence to shorten the deposition time [20]. In the case of atmospheric-pressure spatial ALD methods, precursor vapors are transported via distinct channels to the reactor head, with metal precursors and co-reactant channels isolated from each other by inert gas channels. This configuration prevents precursor reactions above the substrate while a heated moving substrate cycles beneath the gas head and channels [41]. Some laboratories have reported using s-ALD to deposit materials such as nickel oxide (NiO) and SnO_2_ for the hole transport layer and electron transport layer, respectively. Additionally, rapid vapor-phase deposition techniques and AP-CVD methods have proven successful in the integration of buffer layers for semitransparent PSC devices.

Another challenge in implementing ALD interlayers is the long deposition times, which may not be favorable for mass production. A possible solution for this can be pulsed-CVD. As mentioned above, pulsed-CVD is a variation of ALD that is useful for cutting down on the deposition time. Pulsed-CVD involves exposing the two precursors at the same time, instead of separately, and reducing the purging time, which results in substantially reducing the deposition time. Pulsed-CVD can be a promising alternative to ALD for mass production.

## 5. Summary and Future Outlook

ALD tools offer a powerful and versatile approach to depositing high-quality thin films, which can enhance charge collection and stability of perovskite solar cells. However, numerous challenges persist, particularly when incorporating ALD films in layers positioned above the perovskite absorber. These applications include passivation layers at the perovskite surface, barrier or protection layers at the CTL and top metal contact interface, buffer layers in semitransparent and tandem configurations, and encapsulation layers designed to enhance device stability against external degradation factors. ALD delivers pinhole-free, high-quality, and uniform inorganic materials under conditions which can be made compatible with the processing of hybrid devices. It also delivers exceptionally reproducible films and enables precise control of material properties, encompassing doping, stoichiometry, and electrical/optical characteristics. However, several challenges must be addressed for ALD to realize its full potential in advancing perovskite solar cells. These challenges include reducing lengthy deposition times, minimizing damage from ALD precursors, and managing elevated temperatures.

Semitransparent and tandem applications hold great promise for the solar PV industry, as they offer cost-effective pathways to enhance solar cell efficiencies. With commercialization in mind, ALD and its variations, such as pulsed-CVD, AP-CVD, and s-ALD, are poised to play a crucial role in the development of perovskite photovoltaics that demand highly efficient and stable devices for large-area coatings. These innovations also have potential applications in the realm of flexible electronic devices.

## Figures and Tables

**Table 2:** Summary of deposition parameters and equipment on recent literature on ALD interlayers inserted above the absorber in perovskite solar cells.

Material	Precursors	Temp. (°C)	Process	Equipment	Institute, Year [Ref]
Al_2_O_3_	TMA + H_2_O	100	ALD	NCD, Lucida D-100	KRICT, 2023 [21]
Al_2_O_3_	TMA + HPLC Grade H_2_O	70	ALD	Home-made ALD system	IIT Bombay, 2023 [22]
Al_2_O_3_	TMA + H_2_O	100	ALD	Oxford Instrument OpAL^TM^	Eindhoven, 2017 [23]
CuO*_x_*	Cu(dmamb)_2_ + H_2_O	100	Pulsed-CVD	CN-1, Atomic Classic	KRICT, 2020 [20]
CuO*_x_*	ATHFAACu + H_2_O	100	AP-CVD	Vertical Cambridge University Close Proximity (V-CUCP)	Cambridge, 2020 [24]
Ga_2_O_3_	Ga_2_(NMe_2_)_6_ + H_2_O	120	ALD	-	Wuhan, 2018 [25]
SiAl*_x_*O*_y/_*SiO_2_	TEOS, TMA + H_2_O/TEOS + H_2_O	100	ALD	-	NCEPU, 2023 [26]
SnO*_x_*	TDMASn + H_2_O	90	ALD	Beneq TFS-200	NREL, 2023 [27]
SnO_2_	TDMASn + H_2_O_2_	50	ALD	Sentech SE401adv	Nankai U., 2022 [28]
SnO_2_	TDMASn + H_2_O	85	ALD	-	Nankai U., 2022 [29]
SnO_2_	TDMASn + H_2_O	100	ALD	-	UNC, 2019 [30]
SnO_2_	TDMASn + H_2_O	100	ALD	Oxford Instrument	EPFL, 2019 [31]
SnO*_x_*/Zn:SnO*_x_*	TDMASn/DEZ + H_2_O	85	ALD	Beneq TFS-200	NREL, 2019 [32]
*a*-TiO_2_	TDMATi + H_2_O	60	ALD	Home-made ALD system	SKKU, 2021 [18]
V_2_O_5-*x*_	VTIP + H_2_O	45	ALD	Home-made ALD system	SKKU, 2022 [19]
VO*_x_*	VTIP + H_2_O	80	ALD	Arradiance Gemstar-6	Stanford, 2019 [33]
ZrO_2_	TDMAZr + O_3_	80	ALD	LabNano ALD	SCN, 2018 [34]
